# Intraoperative complexity markers are associated with morbidity but not mortality in emergency abdominal surgery: a two-year cohort study

**DOI:** 10.1007/s00423-025-03941-z

**Published:** 2026-01-16

**Authors:** Lasse Rehné Jensen, Klara Thorhauge, Dunja Kokotovic, Thomas Korgaard Jensen, Jakob Burcharth

**Affiliations:** 1 Department of Gastrointestinal and Hepatic Diseases, Copenhagen University Hospital - Herlev and Gentofte, Herlev, Denmark; 2 Department of Clinical Medicine, University of Copenhagen, Copenhagen, Denmark; 3 Emergency Surgery Research Group Copenhagen (EMERGE Cph), Copenhagen University Hospital - Herlev, Herlev, Denmark

**Keywords:** Emergency surgery, Complexity, High-risk patients, Postoperative outcomes, intraoperative complications, Emergency laparotomy

## Abstract

**Purpose:**

Patients undergoing major emergency abdominal surgery are often elderly with multiple comorbidities and previous abdominal operations, contributing to procedural complexity. Factors such as adhesions increase technical challenges, potentially influencing postoperative recovery. This study examined how objectively defined intraoperative complexity markers are associated with postoperative morbidity and mortality in this high-risk cohort.

**Methods:**

In a prospective cohort of 754 consecutive patients undergoing major emergency abdominal procedures, we investigated three indicators of intraoperative complexity: iatrogenic injury, blood loss *≥* 750 mL, and operative duration *≥* 2.5 h. A composite variable incorporating all three was also created to reflect overall complexity. We analyzed associations with postoperative outcomes, including complication severity, length of stay (LOS), reoperations, and mortality.

**Results:**

At least one complexity marker was observed in 32% of patients. Bleeding *≥* 750 mL and prolonged operative time each independently increased the likelihood of extended hospitalization by 23.0 and 22.1% points, respectively. Iatrogenic injuries were identified in 14% and correlated with longer LOS and increased reoperations. Although complexity markers were consistently linked to higher morbidity, including elevated Comprehensive Complication Index scores, reoperations, and prolonged LOS. No significant association with mortality was observed.

**Conclusion:**

Intraoperative complexity is frequent in major emergency abdominal surgery and is closely associated with postoperative morbidity and healthcare utilization. Bleeding exceeding 750 mL and operative time over 2.5 were the strongest associations with postoperative morbidity. These findings provide a pragmatic framework for quantifying surgical complexity and may inform future work on preoperative risk stratification and resource planning. The observed dissociation between morbidity and mortality may reflect improved perioperative care and patient selection, but should be interpreted cautiously given the limited number of deaths.

**Supplementary Information:**

The online version contains supplementary material available at 10.1007/s00423-025-03941-z.

## Introduction

Major emergency abdominal surgery, including procedures for bowel obstruction, perforation, and ischemia, is associated with considerable morbidity and mortality [1–5]. Despite standardized care bundles improving outcomes [6, 7], 30-day mortality remains high, ranging from 8% to 25%, and up to 50% in patients aged 80 or older [6, 8–10]. These patients are typically older, variably frail, and present with diverse surgical histories and comorbidities [1, 3, 6, 11, 12]. Prior abdominal operations are frequent and can alter intra-abdominal conditions through adhesion formation [13]. However, evidence on how surgical history influences intraoperative complexity remains limited. In a recent study by our group, 61% had undergone prior abdominal surgery, with previous colorectal resections particularly linked to more complex procedures [14]. Additionally, a national survey of Danish surgeons highlighted previous open abdomen, difficult prior surgery, advanced liver cirrhosis, prior abdominal or pelvic radiotherapy, and diffuse peritonitis as key contributors to anticipated intraoperative complexity [15].

In contrast to elective hepatopancreatic and laparoscopic procedures, where predictors of technical difficulty are well described [16–19], such data in emergency abdominal surgery remain scarce. These predictors typically correlate with longer operative times, increased blood loss, and higher complication rates [20–23]. Adhesions, particularly after colorectal surgery [24], are a common source of difficulty and increase procedural complexity. Iatrogenic bowel injury occurs in up to 40% of adhesiolysis cases, highlighting the clinical impact of adhesions [13, 25, 26]. Intraoperative complexity usually reflects a combination of technical challenges rather than a single event.

Evidence on contributors to intraoperative complexity in emergency surgery is limited. This study aimed to quantify how objectively defined intraoperative complexity markers, defined as iatrogenic injury, significant bleeding, and prolonged operative time, relate to postoperative morbidity and mortality. Rather than focusing on preoperative prediction, our objective was to characterize the clinical impact of intraoperative complexity once encountered, thereby providing a foundation for future predictive and risk-stratification research.

## Materials and methods

This study followed the STROBE guidelines for observational research [27]. Approvals were obtained from the Capital Region of Denmark (P-2020-1166, R-21038079) and the Danish Data Protection Agency (P-2021-431). Ethical approval was not required under Danish law due to the study’s non-interventional design.

### Data source and population

The study cohort included both patients presenting with new-onset abdominal emergencies (e.g., perforation, ischemia, or obstruction) and patients requiring emergency reoperation for major postoperative complications such as anastomotic leakage or intra-abdominal sepsis, all of whom are managed within the standardized AHA protocol. The study was conducted at a single Danish university hospital (catchment ~ 465,000) from January 1, 2021, to December 31, 2022. While most procedure- and patient-related variables were prospectively registered in our institutional database, surgical history and comorbidity data were retrospectively extracted from medical records and entered into REDCap. Ambiguities were clarified with senior surgeons. Variables included demographics, intraoperative details (surgical intent, findings, interventions, adhesions, carcinomatosis, contamination), prior surgeries, and intraoperative outcomes (iatrogenic injury, blood loss, duration). Comorbidity data were obtained from patient records and the regional electronic health record system (EPIC), which captures all physician-registered diagnoses and prescriptions.

The hospital uses a multidisciplinary perioperative protocol, AHA (Acute High-risk Abdominal surgery), for patients undergoing emergency abdominal surgery due to severe intra-abdominal disease. The protocol spans the pre-, intra-, and postoperative phases [6, 9, 29–33]. It emphasizes early assessment, CT within 2 h, and surgery within 6 h. Intraoperative care includes structured team decisions, time-outs, standardized anesthetic management, and predefined criteria for surgical approach (definitive, palliative, or damage control). Postoperatively, care is stratified by condition and includes ICU/high-dependency monitoring, daily senior rounds, nursing plans, early dietician-led nutrition, and physiotherapy-supported mobilization. The full protocol is available in Supplementary Material. Denmark has a publicly funded, tax-based healthcare system providing universal coverage without user payment. Emergency abdominal procedures such as bowel obstruction, perforation, or ischemia are exclusively performed in public hospitals. Surgical training comprises six years, of which the final five constitute the residency period. Trainees typically reach senior registrar level after 2–3 years and often serve as the most senior surgeon in-house. Emergency surgery is a recognized subspecialty within gastrointestinal surgery, and most departments maintain dedicated emergency teams. Consultant surgeons provide continuous 24/7 coverage for acute surgical conditions, either on-site during daytime or on call with immediate availability during evenings and nights.

At our institution, patients with severe intra-abdominal pathology have access to high-dependency and intensive care units led by specialized surgical and anesthesiology teams. Interventional radiology is available during daytime hours, while emergency cases outside regular hours can be transferred within 30 min to a tertiary referral center for urgent image-guided intervention. This system ensures continuous access to critical care and advanced interventional support for patients requiring complex emergency management.

A definitive approach was defined as curative surgery addressing the primary pathology. Palliative procedures aimed to relieve symptoms without curative intent, e.g., diverting stoma or bypass in malignant obstruction. Damage control surgery (DCS) referred to an abbreviated intervention for bleeding or contamination in unstable patients, with definitive treatment deferred until stabilization [33].

Adhesions were graded using Zühlke’s classification: grade 0 (none), grade 1 (filmy, bluntly separable), grade 2 (sharp dissection, early vascularization), grade 3 (dense, vascularized, requiring sharp dissection), and grade 4 (frozen abdomen with firm organ attachments and high injury risk) [34].

### Outcome measures

The primary outcome was the association between predefined intraoperative complexity markers and postoperative events. Based on literature, expert input, and case reviews, three key indicators were identified:

1. Iatrogenic lesions, including unintended injuries such as serosal tears, full-thickness bowel perforations, and solid organ damage: These are commonly linked to adhesiolysis in bowel obstruction, and associated with longer operative times, complications, reoperations, and readmissions [35, 36].
2. Procedure time ≥ 2.5 h: Longer procedures generally reflect increased technical difficulty due to advanced dissection or reconstruction, with complication rates rise beyond 2.1 h and peak at 6.4 h [37]. The 2.5-hour cutoff was chosen to capture complex cases while excluding moderate ones.
3. Intraoperative bleeding ≥ 750 mL: This threshold was selected as a clinically relevant marker. A scoping review identified intraoperative blood loss as a commonly used complexity measure across surgical classification systems [38]. In trauma literature, Advanced Trauma Life Support (ATLS) defines Class II hemorrhage as 750–1,500 mL, with expected physiological consequences [39]. Increased intraoperative blood loss has been demonstrated in complex compared to non-complex pancreatoduodenectomy procedures, supporting its role as a marker of surgical complexity [40].

These markers align with previous classification systems [19, 23, 38]. An earlier publication on this topic, focusing on high-risk complexity markers, chose procedural time *≥* 3 h and excessive bleeding *≥* 1000 mL, respectively [14]. For the present analyses, we deliberately lowered these thresholds to 2.5 h and 750 mL to better reflect clinically relevant cut-offs and enhance the applicability of our findings to everyday surgical practice. To statistically support these predefined thresholds, receiver operating characteristic (ROC) analyses were performed for both intraoperative bleeding and operative time against key postoperative outcomes (length of stay, reoperation, severe complications, and mortality). Optimal cut-offs were determined using Youden’s index. The results consistently supported the predefined limits of 750 mL and 2.5 h as clinically and statistically justified markers of intraoperative complexity. Detailed ROC metrics are presented in Supplementary Tables 5 and 6.

Postoperative outcomes included Comprehensive Complication Index (CCI) [41], length of hospital stay (LOS), prolonged LOS (>7 days), severe complications (Clavien-Dindo *≥* 3b) [42], reoperation during the index admission, and mortality (30-day and in-hospital). Depending on clinical relevance, outcomes were evaluated as continuous (CCI and LOS) and binary variables. All outcome analyses were adjusted for relevant confounders, including age, sex, American Society of Anesthesiologists-score (ASA) >2, chronic obstructive pulmonary disease (COPD), cirrhosis, diabetes, chronic kidney disease (CKD), and previous myocardial infarction (MI) or ischemic heart disease (IHD).

### Statistical analyses

Categorical variables are presented as counts and percentages, continuous variables as medians with interquartile ranges (IQR). Data distribution was checked via histograms and Shapiro-Wilk test. Descriptive analyses included frequencies and overlaps of complexity markers. Bivariate comparisons were conducted using chi-square tests for categorical and Mann-Whitney U tests for continuous variables. Graphical outputs included bar charts for marker frequencies, grouped bar charts (chi-square tests) for categorical comparisons, line graphs (Mann-Whitney U) for CCI distributions, and a radar plot illustrating relative impact across outcomes.

Primary associations between intraoperative complexity markers (iatrogenic lesion, procedure time *≥* 2.5 h, bleeding *≥* 750 mL, and a composite complexity measure) and postoperative outcomes were assessed using multivariable logistic regression for binary outcomes and linear regression for continuous outcomes. Results are reported as odds ratios (OR) or beta coefficients (β) with 95% confidence intervals (CI). All models were adjusted for age, sex, ASA > 2, COPD, cirrhosis, diabetes, CKD, and prior MI or IHD. For continuous outcomes, β-coefficients indicate the adjusted mean difference. To identify independent predictors of 30-day mortality, multivariable logistic regression was performed, including demographics, comorbidities, intraoperative pathology and complexity markers. In supplementary analyses, ROC curves were constructed for intraoperative bleeding and operative time to assess their discriminatory ability for postoperative outcomes, with optimal thresholds identified by Youden’s index.

Average Marginal Effects (AMEs) were calculated to improve interpretability, estimating the absolute change in outcome probability or mean difference associated with each marker. AMEs were derived using the delta method with identical covariate adjustment. Model diagnostics included variance inflation factors (VIF < 5), Hosmer-Lemeshow tests for logistic models, and residual plots for linear models. Complete case analysis was applied; missing data were minimal (< 3% for key variables, except bleeding volume at 3%). Statistical significance was defined as *p* < 0.05 (two-tailed). The analyses were conducted in R (version 2025.09.1 + 401).

## Results

### Patient characteristics

We included 754 patients undergoing major emergency abdominal surgery. The median age was 71 years (IQR: 58–79), and 51% were female. The mean BMI was 25 (SD ± 5), 40% had ASA > 2, and 26% had a performance status > 1. Common comorbidities included diabetes (11%), previous MI and/or IHD (9%), COPD (8%), liver cirrhosis (3%), and chronic kidney disease (3%). A total of 460 patients (61%) had a history of abdominal and/or pelvic surgery, including 249 (33%) with previous open surgery, 108 (14%) with laparoscopic procedures only, and 97 (13%) with both laparoscopic and open surgery. The surgeon in charge was a senior registrar in 341 cases (45%), a senior consultant in 219 cases (29%), and a resident in 183 cases (24%). In 11 cases (1%), the responsible surgeon was not specified. The surgical approach was laparotomy in 386 cases (51%), laparotomy converted from laparoscopy in 208 cases (28%), and primary laparoscopy in 160 cases (21%). Data are outlined in Supplementary Table 1. The overall median follow-up time for mortality was 439 days (IQR: 325–634). An overview of the detailed intraoperative findings and procedures can be found in Supplementary Table 2.

### Iatrogenic lesions

Iatrogenic lesions occurred in 104 patients (14%). The most frequent injuries were serosal lesions to the small bowel (39%) followed by full-thickness perforations of the small bowel (38%). Less common injuries included serosal lesions to the colon (7%), full-thickness colonic perforations (2%), and lesions to the spleen (5%) or liver (1%). Other lesions accounted for 16% and comprised mesentery, gallbladder, common bile duct, and blood vessels. Patients with iatrogenic injuries had a significantly longer postoperative hospital stay (β: 7.17, 95% CI: 3.70–10.64) and higher odds of prolonged admission (> 7 days, OR: 1.72, 95% CI: 1.10–2.69). Iatrogenic lesions were also associated with an increased need for reoperation during admission (OR: 1.86, 95% CI: 1.12–3.09), but not with severe complications (CD *≥* 3b), 30-day mortality, or overall mortality (Table 1). Patients with non-serosal iatrogenic injuries (*n* = 68) had a significantly longer postoperative hospital stay (β: 10.8, 95% CI: 6.7–14.9), higher odds of prolonged hospitalization (> 7 days; OR: 2.20, 95% CI: 1.29–3.80), and increased risk of reoperation during admission (OR: 2.14, 95% CI: 1.16–3.80). A supplementary analysis was limited to serosal defects only (*n* = 48). These were not associated with longer postoperative hospital stay (β = 0.05, 95% CI: − 4.9–5.0), prolonged hospitalization (> 7 days; OR = 1.27, 95% CI: 0.67–2.40), or reoperation (OR = 1.00, 95% CI: 0.42–2.11).

**Table 1:** Associations between markers of intraoperative complexity and postoperative outcomes

Postoperative course	*N* = 754 (%)	Iatrogenic lesions *n* = 104 (14% of tot.)	Procedure time *≥* 2.5 *n* = 168 (22% of tot.)	Bleeding *≥* 750 mL *n* = 56 (7% of tot.)	Complex procedure *n* = 243 (32% of tot.)
		β (CI)	β (CI)	β (CI)	β (CI)
**CCI** **Length of stay**	Median = 20.90 (IQR: 0–42.40) Median = 7 (IQR: 4–12)	4.52 (-1.50–10.55) **7.17 (3.70–10.64)**	**5.90 (0.88–10.93)** **7.11 (4.27–9.96)**	4.83 (-3.29–12.95) **9.72 (5.09–14.35)**	**5.91 (1.43–10.38)** **5.67 (3.10–8.24)**
		OR (95% CI)	OR (95% CI)	OR (95% CI)	OR (95% CI)
**Length of stay**,** > 7 days** **CD** **≥** **3b** **Reoperation during admission** **Mortality**,** 30-day** **Overall mortality**	*n* = 324 (43%) *n* = 165 (22%) *n* = 131 (17%) *n* = 87 (12%) *n* = 223 (31%)	**1.72 (1.10–2.69)** 1.49 (0.92–2.40) **1.86 (1.12–3.09)** 0.76 (0.37–1.54) 0.80 (0.46–1.37)	**2.78 (1.90–4.08)** 1.40 (0.93–2.10) **1.68 (1.09–2.61)** 0.64 (0.34–1.20) 0.99 (0.63–1.56)	**2.49 (1.34–4.63)** 1.38 (0.74–2.56) **2.39 (1.28–4.48)** 0.66 (0.24–1.82) 1.10 (0.54–2.23)	**2.55 (1.81–3.59)** **1.44 (1.00–2.07)** **1.95 (1.31–2.92)** 0.68 (0.39–1.17) 1.03 (0.69–1.55)

Associations between four predefinedmarkers of intraoperative complexity, iatrogenic lesions, procedure time >2.5 hours, intraoperativebleeding >750 mL, and complex procedure, and postoperative outcomes are presented. Forcontinuous outcomes, β coefficients with 95% confidence intervals (CI) are reported; for binaryoutcomes, odds ratios (OR) with 95% CI are shown. Statistically significant associations (p < 0.05)are indicated in bold. β coefficients represent the mean difference in outcome between patients withand without the given intraoperative factor, adjusted for confounders. For example, patientsundergoing a complex procedure had a significantly higher Comprehensive Complication Index(CCI), with an adjusted β of 5.91 (95% CI: 1.43–10.38), indicating an average CCI increase of 5.91 points compared to patients without complexity. All analyses were adjusted for age, sex, ASA >2,COPD, cirrhosis, diabetes, CKD, and previous MI or IHD

### Prolonged procedure time

Procedures exceeding two and a half hours (*n* = 168, 22%) were associated with a significantly longer length of stay (β: 7.11, 95% CI: 4.27–9.96) and higher odds of prolonged admission (OR: 2.78, 95% CI: 1.90–4.08). They were also associated with significantly increased odds of reoperation (OR: 1.68, 95% CI: 1.09–2.61). No associations were found with mortality outcomes (Table 1).

### Major bleeding

Intraoperative bleeding above 750 mL (*n* = 56, 7%) was linked to more extended hospital stays (β: 9.72, 95% CI: 5.09–14.35) and increased odds of admission > 7 days (OR: 2.49, 95% CI: 1.34–4.63). Major bleeding was significantly associated with higher odds of reoperation (OR: 2.39, 95% CI: 1.28–4.48). No significant associations were found with major complications or mortality (Table 1).

### Complex procedure (composite outcome)

The composite complexity variable marker (presence of any of: iatrogenic injury, blood loss *≥* 750 mL, or operative time *≥* 2.5 h) was present in 32% of patients (243/754, Supplementary Fig. 1) and was associated with increased morbidity: longer postoperative stays (β: 5.67, 95% CI: 2.67–8.24), higher odds of prolonged admission (OR: 2.55, 95% CI: 1.81–3.49), severe complications (OR: 1.44, 95% CI: 1.00–2.07), and reoperation (OR: 1.95, 95% CI: 1.31–2.92). There was no significant association with mortality outcomes (Table 1).

### Distribution of comprehensive complication index scores by complexity status

**Fig. 1:**
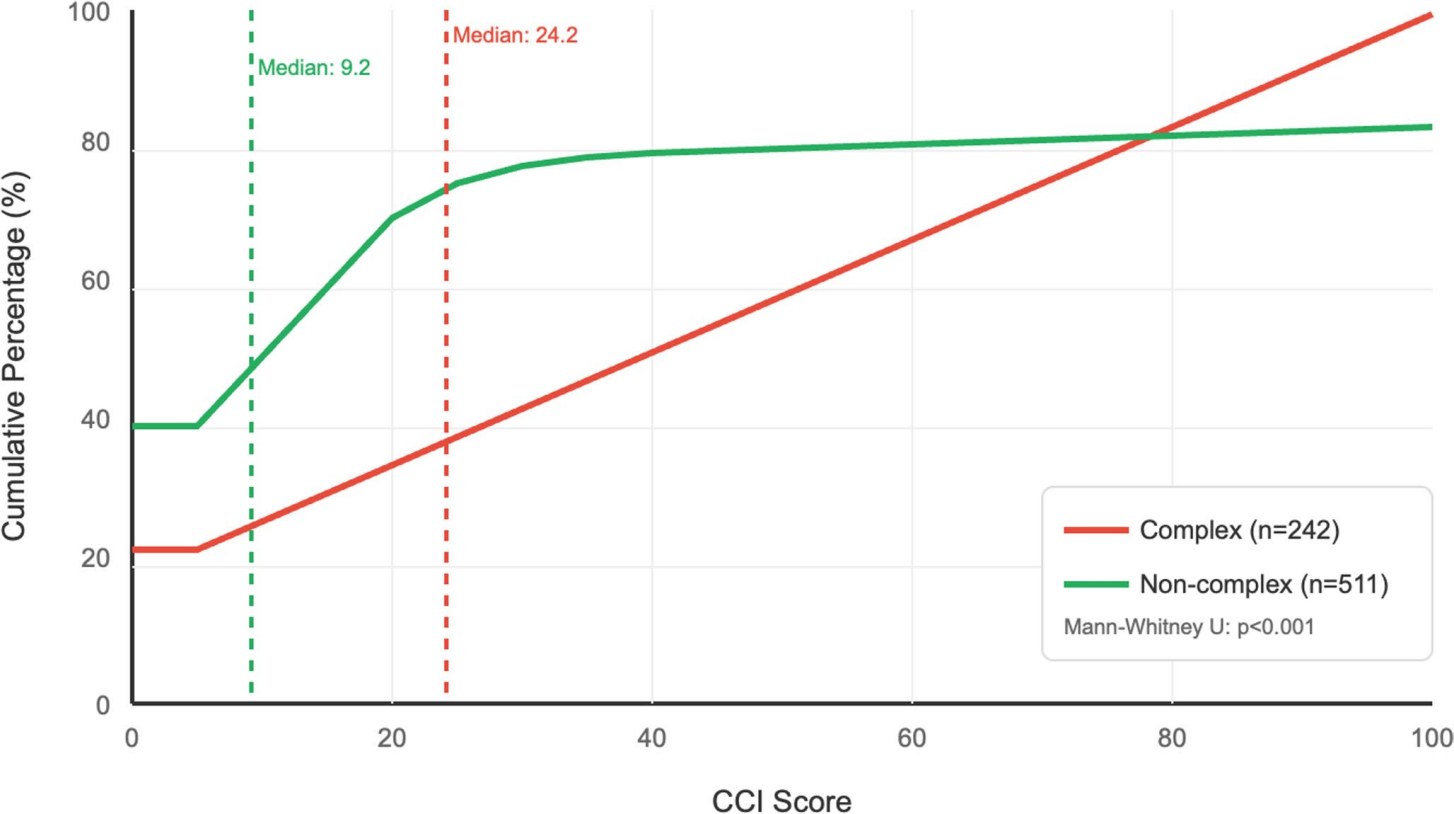
Distribution of comprehensive complication Index (CCI) scores by intraoperative complexity status. The cumulative distribution curves show patients with complex procedures (*n*=243) had a rightward shift compared to those without complexity (*n*=511), with higher median CCI scores (24.2 vs 9.2, Mann-Whitney U test, *p*<0.001). The divergent distributions illustrate the increased cumulative morbidity burden associated with intraoperative complexity

Patients undergoing complex procedures (*n* = 243, 32%) showed a rightward shift in the CCI distribution compared to those without complexity (*n* = 511), reflected in higher median scores (24.2 vs. 9.2; Mann-Whitney U test, *p* < 0.001), as shown in Fig. 1. This divergence highlights the greater cumulative morbidity burden associated with intraoperative complexity. To illustrate the distribution underlying this cumulative difference, Supplemental Fig. 2 displays the frequency distribution of CCI scores across 10-point intervals. Complex cases were more evenly distributed across the CCI spectrum, while non-complex cases clustered at lower scores (0–19). This explains the linear cumulative curve for complex cases in Fig. 1 and supports a biologically meaningful difference in postoperative morbidity.

### Comparative impact profile

**Fig. 2:**
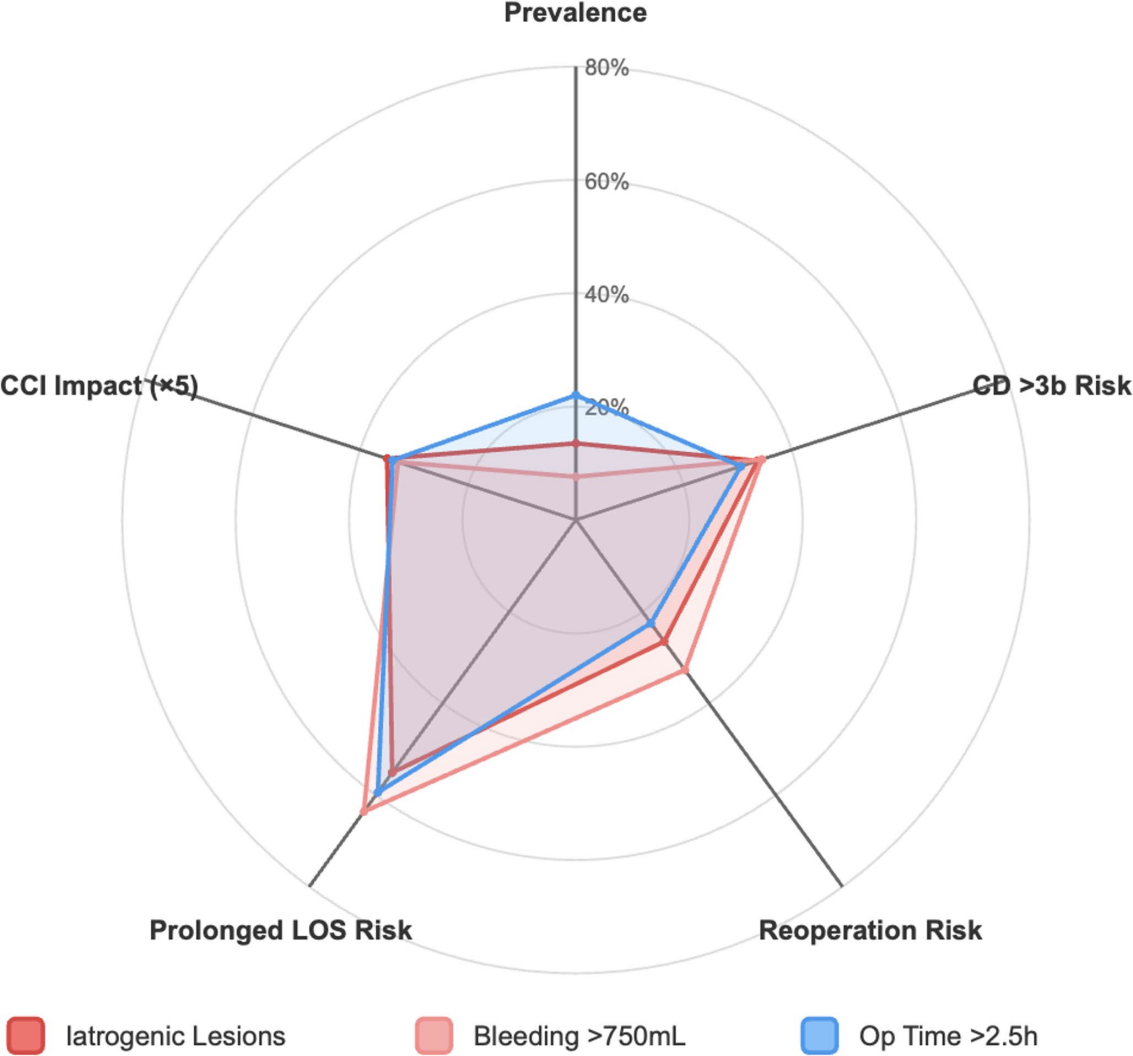
Relative impact profile of intraoperative complexity markers across key postoperative outcomes. Radar plot comparing iatrogenic lesions, intraoperative bleeding >750 mL, and operative time >2.5 hours across five domains: prevalence, risk of Clavien-Dindo >3b complication, reoperation, prolonged length of stay (LOS >7 days), and mean Comprehensive Complication Index (CCI) increase. Prevalence is included solely to illustrate the relative frequency of each complexity marker and to contextualize their respective outcome impacts. Operative time >2.5 hours showed the highest overall prevalence (22.0%) and consistent impact across outcomes. Bleeding >750 mL, despite lower prevalence (7.6%), was strongly associated with prolonged hospitalization (63.6%) and reoperation (32.7%). All three markers showed similar CCI impact (6.6–7.0 points, scaled ×5 for visualization). Iatrogenic injuries demonstrated moderate, consistent effects across all outcomes and may serve as a balanced indicator of intraoperative technical challenge

A radar plot comparing iatrogenic lesions, intraoperative bleeding *≥* 750 mL, and operative time *≥* 2.5 h across four domains: risk of CD *≥* 3b complication, reoperation, prolonged length of stay (LOS > 7 days), and mean CCI increase (scaled ×5 for visualization). Operative time *≥* 2.5 h showed the highest overall prevalence (22.0%) and consistent impact across outcomes. Bleeding *≥* 750 mL, despite lower prevalence (7.6%), was strongly associated with prolonged hospitalization (63.6%) and reoperation (32.7%). All three markers showed similar CCI increases (6.6–7.0 points), while iatrogenic injuries demonstrated moderate, consistent effects across all domains (Fig. 2).

### Average marginal effects

In the AME analyses, bleeding *≥* 750 mL and operating time *≥* 2.5 h were most strongly associated with prolonged hospitalization, increasing the probability of LOS > 7 days by 23.0 (95% CI: 8.5–37.5, *p* = 0.002) and 22.1% points (95% CI: 12.8–31.4, *p* < 0.001), respectively. The composite complexity measure (+ 17.4 pp, *p* < 0.001) and iatrogenic injuries (+ 12.6 pp, *p* = 0.01) also showed significant effects. Bleeding, iatrogenic injuries, and the composite measure were further associated with increased reoperation risk (Supplementary Table 3).

For continuous outcomes, all complexity markers were associated with significantly longer hospital stays. The most pronounced effects were seen for bleeding *≥* 750 mL (+ 9.7 days, *p* < 0.001), operating time *≥* 2.5 h (+ 8.9 days, *p* < 0.001), and iatrogenic injuries (+ 7.2 days, *p* < 0.001). The composite measure increased LOS by 5.7 days (*p* < 0.001). CCI scores were significantly higher for operating time *≥* 2.5 h (+ 6.5 points, *p* = 0.03) and the composite measure (+ 6.4 points, *p* = 0.009) (Supplementary Table 3).

Notably, no complexity marker increased 30-day mortality. All showed negative AMEs ranging from − 6.8 to 7.1% points (all *p* > 0.05) (Supplementary Tables 3–4).

### 30-day mortality

**Fig. 3:**
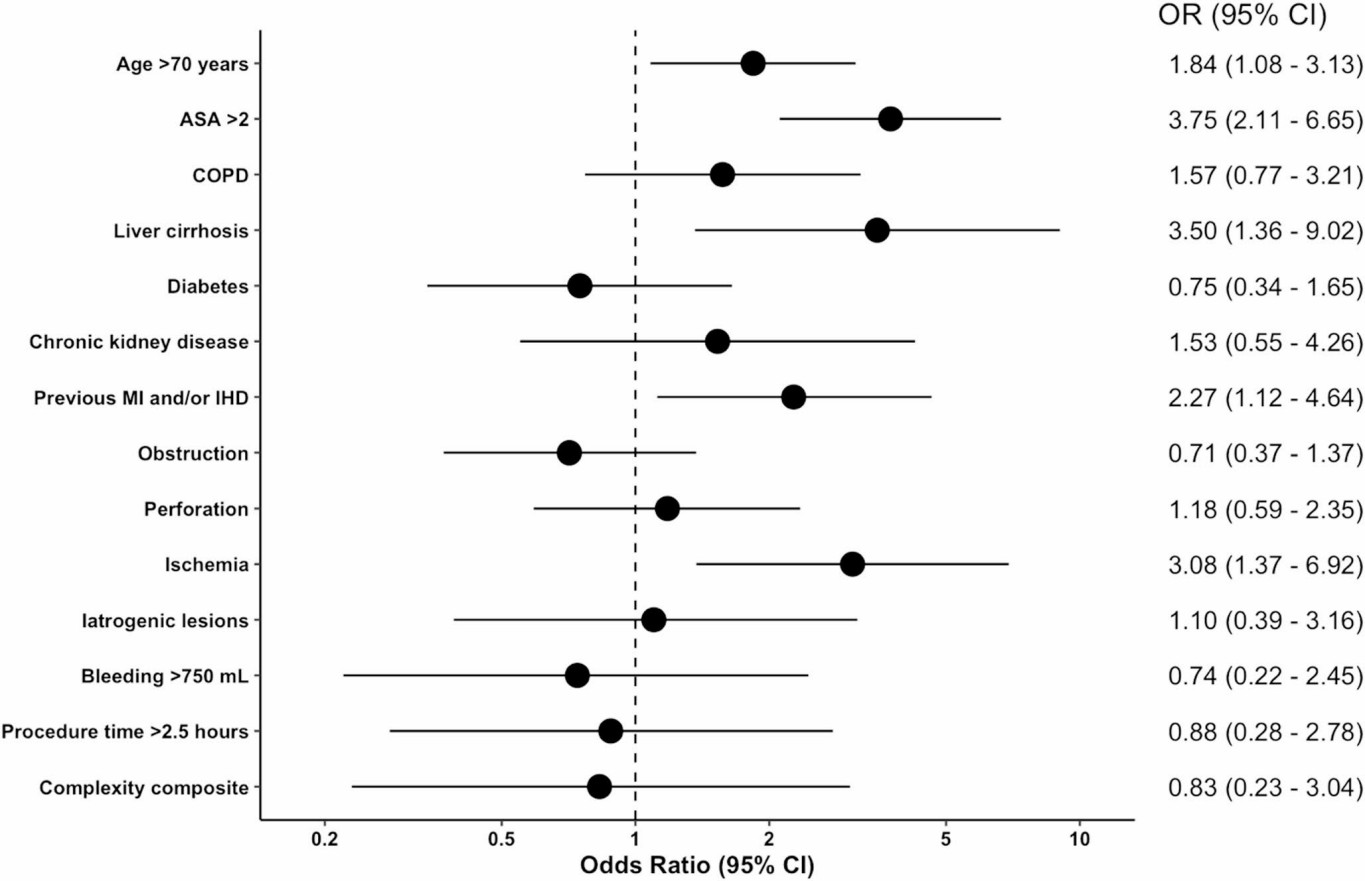
Multivariate logistic regression analysis of demographic, intraoperative and complexity risk factors for 30-day mortality. ASA; American Society of Anesthesiologists, COPD; Chronic obstructive pulmonary disease, IHD; Ischemic heart disease, MI; Myocardial infarction OR; Odds ratio.

In the multivariate logistic regression analysis, age *≥* 70 years (OR: 1.95, 95% CI: 1.11–3.43, *p* = 0.02), ASA score > 2 (OR: 3.75, 95% CI: 2.11–6.65, *p* < 0.001), liver cirrhosis (OR: 3.50, 95% CI: 1.36–9.02, *p* = 0.01), and a history of myocardial infarction and/or ischemic heart disease (OR: 2.27, 95% CI: 1.12–4.64, *p* = 0.02) were all independently associated with increased 30-day mortality. Additionally, intraoperative intestinal ischemia was a significant predictor (OR: 3.08, 95% CI: 1.37–6.92, *p* = 0.006). Other comorbidities and complexity-related factors, including bleeding, prolonged procedure time, and iatrogenic injury, were not significantly associated with mortality (Fig. 3).

## Discussion

In this prospective cohort study of 754 patients undergoing major emergency abdominal surgery, intraoperative complexity markers were present in 32% of cases and were consistently associated with increased postoperative morbidity, though not mortality. Bleeding *≥* 750 mL and operative time *≥* 2.5 h had the greatest impact, increasing the probability of prolonged hospitalization by 23.0 and 22.1% points, respectively. This equates to 23 of 100 patients with major bleeding and 22 with prolonged procedures requiring extended hospital stays, important for resource allocation and preoperative counselling. The lack of association between intraoperative complexity and mortality likely reflects a combination of limited statistical power due to relatively few death events and potential selection effects, where frail or terminal patients are less likely to undergo extensive procedures. These findings describe how intraoperative technical challenges, once encountered, influence postoperative recovery and healthcare utilization. The study does not aim to predict complexity preoperatively but rather to quantify its downstream clinical impact.

There is currently no established method for quantifying intraoperative complexity in emergency abdominal surgery. In this study, we focused on the most frequent unintended intraoperative events, organ injury, and significant bleeding, as well as prolonged procedural time, which may reflect technically challenging conditions. The present definition of intraoperative complexity was limited to objectively measurable intraoperative events and should be regarded as a pragmatic construct rather than a comprehensive definition. Future research should aim to integrate procedural, anatomical, and team-based dimensions to refine the conceptual understanding of surgical complexity in emergency settings.

Iatrogenic lesions occurred in 14% of the patients and were significantly associated with prolonged hospital stay and increased risk of reoperation during admission. Although no significant association was observed with 30-day mortality or overall complication severity, these injuries remain clinically essential indicators of intraoperative difficulty. The majority involved the small bowel, likely reflecting the technical challenges encountered during emergency abdominal procedures. Such lesions involving hollow organs, vessels, or solid structures like the spleen are frequently linked to adhesiolysis, a well-established risk factor for intraoperative injury, delayed recovery, and increased procedural complexity [14, 25, 35, 36]. An exploratory subgroup analysis limited to serosal defects showed no association with postoperative morbidity or reoperation. This finding suggests that serosal lesions alone may not independently increase postoperative risk but likely represent minor components of technically demanding adhesiolysis. Their inclusion within the broader iatrogenic injury category was maintained to preserve the predefined complexity construct and to reflect the frequent coexistence of such minor injuries with other intraoperative challenges.

7% of patients in our cohort experienced intraoperative bleeding exceeding 750 mL. This volume was significantly associated with prolonged length of stay and reoperations but not increased mortality. Although peri- and postoperative transfusions in non-cardiac surgery, including major emergency abdominal procedures, have consistently been associated with higher rates of postoperative complications and mortality [43–45], this was not observed in our data. One possible explanation is that a blood loss of approximately 750 mL may not be sufficient, by itself, necessitate transfusion in otherwise stable adult patients. Clinical guidelines recommend a restrictive transfusion threshold of 7.0–9.0 g/dL hemoglobin in stable individuals without cardiovascular disease, corresponding to a blood loss closer to 30% of total blood volume, roughly 1,500 mL or more in an average adult, before transfusion becomes necessary [46–48]. Moreover, intraoperative transfusion thresholds are not solely determined by the volume of blood lost but also by patient-specific factors such as hemodynamic stability, comorbidity, and baseline hematocrit [48]. Therefore, our predefined bleeding cut-off may have been too low to induce sufficient physiological stress or hemodynamic compromise to impact postoperative mortality. Nevertheless, bleeding beyond 750 mL was independently associated with prolonged hospitalization and reoperations, supporting its relevance as a marker of increased procedural complexity.

Procedures lasting more than 2.5 h were significantly associated with both higher CCI scores, reoperations, and prolonged length of stay. This aligns with existing literature, where prolonged operative time in abdominal surgery has been linked to a range of complications, including surgical site infections, sepsis, bowel obstruction, wound dehiscence, intra-abdominal abscesses, bleeding, pneumonia, urinary tract infections, and renal failure [49, 50]. In emergency settings specifically, procedures exceeding two hours have been shown to increase the risk of postoperative morbidity and mortality markedly [45]. Several factors may contribute to prolonged procedures, including abdominal adhesions, which are known to increase operative time [13]. Similarly, previous peritonitis, intra-abdominal abscesses, or inflammation, often seen in patients undergoing appendectomy or cholecystectomy, have been associated with longer surgical duration, likely due to adhesion formation and technically challenging dissection [13, 51]. In the context of major emergency abdominal surgery, prolonged operative time reflects increased intraoperative complexity and carries important clinical implications due to its association with adverse postoperative outcomes.

The composite outcome *Complex procedure*, encompassing iatrogenic injury, excessive bleeding, and prolonged operative time, was independently associated with several key markers of adverse postoperative outcomes, including higher complication severity (CCI), longer LOS, increased reoperations, and higher CD. Importantly, no association was found with short- or long-term mortality after adjusting for confounders. This suggests that while intraoperative complexity markedly influences postoperative recovery and resource use, it does not independently predict mortality in this high-risk population.

The main strength of this study is the inclusion of prospectively collected data from all consecutive, unselected patients undergoing major emergency abdominal surgery over two years. The large sample size enabled detection of associations despite population heterogeneity. Another strength is the availability of detailed preoperative and intraoperative data potentially influencing outcomes.

Limitations include the single-center design, which reflects local practices. Notably, our department does not handle highly specialized HPB or upper GI emergency procedures, limiting generalizability to these subgroups. The observational nature precludes causal inference. Although the sample size was substantial, the relatively low number of mortality events limits statistical power to detect modest differences. Therefore, the absence of a mortality association should be interpreted with caution. Similarly, although the sample size was substantial, the low number of major bleeding events reduced power to identify predictors of this outcome. Neither the surgical approach (laparoscopic, open, or converted) nor the degree of peritonitis was included as covariates in the regression models. Both factors may influence postoperative morbidity and length of stay and should be considered in future studies. We chose conservative thresholds to enhance sensitivity and align with clinical decision-making. Higher thresholds risk excluding relevant cases and limiting generalizability. Importantly, the revised thresholds remain consistent with existing literature on intraoperative risk stratification as described above. Although this study included extended follow-up for mortality, data on long-term morbidity such as hernia formation, reoperation, or functional recovery were not available. Future studies should address these outcomes to better capture the lasting impact of intraoperative complexity.

Our findings provide an empirical description of the relationship between intraoperative complexity and postoperative outcomes. While the markers identified here are intraoperative by definition, they establish measurable endpoints that can inform future efforts to develop preoperative prediction tools and benchmark intraoperative performance. This observational framework thus complements ongoing initiatives to enhance risk-adjusted quality assessment and perioperative planning. The high prevalence of complexity (32% of cases) likely creates institutional preparedness that protects against mortality, most likely due to enhanced monitoring, heightened intraoperative vigilance, competence, and automatic escalation to intensive care. This multi-layered response system appears remarkably effective at rescuing patients from potentially fatal complications, as evidenced by the 23-percentage point increase in prolonged hospitalization without corresponding mortality increase. The observed dissociation between morbidity and mortality may reflect improvements in perioperative care, multidisciplinary monitoring, and timely escalation of treatment. Although this could indicate an effective rescue capacity, the current data cannot confirm causality or institutional performance differences. These findings instead highlight the importance of recognizing and documenting intraoperative complexity when evaluating outcomes and allocating postoperative resources.

## Conclusion

In this cohort of 754 emergency abdominal surgeries, intraoperative complexity markers were present in 32% of cases and significantly increased morbidity and resource utilization but not mortality. Bleeding over 750 mL and operating time exceeding 2.5 h emerged as the strongest predictors, increasing the probability of prolonged hospitalization by over 22% points each. These findings suggest that selected intraoperative events can serve as pragmatic indicators of surgical complexity and may support future efforts in risk-adjusted outcome evaluation and resource planning. The observed dissociation between morbidity and mortality may reflect improvements in perioperative care and patient selection; however, this finding should be interpreted cautiously given the limited number of deaths.

## Supplementary Information

Below is the link to the electronic supplementary material.

Supplementary Material 1

Supplementary Material 2

Supplementary Material 3

## Data Availability

Data are available from the corresponding author upon reasonable request.

## References

[1] Tolstrup M-B, Watt SK, Gögenur I (2017) Morbidity and mortality rates after emergency abdominal surgery: an analysis of 4346 patients scheduled for emergency laparotomy or laparoscopy. Langenbeck’s archives of surgery. Germany 402:615–623. 10.1007/s00423-016-1493-1 10.1007/s00423-016-1493-1 27502400

[2] Tengberg LT, Cihoric M, Foss NB, Bay-Nielsen M, Gögenur I, Henriksen R et al (2017) Complications after emergency laparotomy beyond the immediate postoperative period - a retrospective, observational cohort study of 1139 patients. Anaesth Engl 72:309–316. 10.1111/anae.13721 10.1111/anae.13721 27809332

[3] Saunders DI, Murray D, Pichel AC, Varley S, Peden CJ (2012) Variations in mortality after emergency laparotomy: the first report of the UK emergency laparotomy network. Br J Anaesth England: UK Emerg Laparotomy Netw 109:368–375. 10.1093/bja/aes165 10.1093/bja/aes165 22728205

[4] Rehné Jensen L, Snitkjær C, Kokotovic D, Korgaard Jensen T, Burcharth J Understanding early deaths after major emergency abdominal surgery: an observational study of 754 patients. World J Surg. n/a. 10.1002/wjs.12254 10.1002/wjs.12254 38886168

[5] Barrow E, Anderson ID, Varley S, Pichel AC, Peden CJ, Saunders DI et al (2013) Current UK practice in emergency laparotomy. Annals of the Royal college of surgeons of England. England 95:599–603. 10.1308/rcsann.2013.95.8.599 10.1308/003588413X13629960048433 PMC4311539 24165345

[6] Tengberg LT, Bay-Nielsen M, Bisgaard T, Cihoric M, Lauritsen ML, Foss NB (2017) Multidisciplinary perioperative protocol in patients undergoing acute high-risk abdominal surgery. Br J Surg England: AHA Study Group 104:463–471. 10.1002/bjs.10427 10.1002/bjs.10427 28112798

[7] Chana P, Joy M, Casey N, Chang D, Burns EM, Arora S et al (2017) Cohort analysis of outcomes in 69 490 emergency general surgical admissions across an international benchmarking collaborative. BMJ Open Engl 7:e014484. 10.1136/bmjopen-2016-014484 10.1136/bmjopen-2016-014484 PMC5353261 28274969

[8] Oliver CM, Bassett MG, Poulton TE, Anderson ID, Murray DM, Grocott MP et al (2018) Organisational factors and mortality after an emergency laparotomy: multilevel analysis of 39 903 National emergency laparotomy audit patients. Br J Anaesth England: Natl Emerg Laparotomy Audit Collaborators 121:1346–1356. 10.1016/j.bja.2018.07.040 10.1016/j.bja.2018.07.040 30442263

[9] Huddart S, Peden CJ, Swart M, McCormick B, Dickinson M, Mohammed MA et al (2015) Use of a pathway quality improvement care bundle to reduce mortality after emergency laparotomy. Br J Surg England: ELPQuiC Collaborator Group 102:57–66. 10.1002/bjs.9658 10.1002/bjs.9658 PMC4312892 25384994

[10] Gormsen J, Kokotovic D, Jensen TK, Burcharth J (2025) Trends in clinical outcomes after major emergency abdominal surgery in Denmark, data from 2002–2022. JAMA Surg e250858. 10.1001/jamasurg.2025.0858 10.1001/jamasurg.2025.0858 PMC12019674 40266626

[11] Parmar KL, Law J, Carter B, Hewitt J, Boyle JM, Casey P et al (2021) Frailty in Older Patients Undergoing Emergency Laparotomy: Results From the UK Observational Emergency Laparotomy and Frailty (ELF) Study. Ann Surg. United States: ELF Study Group; 273:709–18. 10.1097/SLA.0000000000003402 10.1097/SLA.0000000000003402 31188201

[12] Snitkjær C, Rehné Jensen L, Í Soylu L, Hauge C, Kvist M, Jensen TK et al (2024) Impact of clinical frailty on surgical and non-surgical complications after major emergency abdominal surgery. BJS Open Engl 8. 10.1093/bjsopen/zrae039 10.1093/bjsopen/zrae039 PMC11126315 38788680

[13] ten Broek RPG, Issa Y, van Santbrink EJP, Bouvy ND, Kruitwagen RFPM, Jeekel J et al (2013) Burden of adhesions in abdominal and pelvic surgery: systematic review and met-analysis. BMJ (Clinical research ed). England 347:f5588. 10.1136/bmj.f5588 10.1136/bmj.f5588 PMC3789584 24092941

[14] Rehné Jensen L, Thorhauge K, Kokotovic D, Jensen TK, Burcharth J (2025) Patients’ surgical history profile and its association with complexity in major emergency abdominal surgery. J Surg Res 310:57–67. 10.1016/j.jss.2025.03.051 40273734 10.1016/j.jss.2025.03.051

[15] Jensen LR, Kokotovic D, Gormsen J, Burcharth J, Jensen TK (2025) Surgeon perspectives on factors affecting intraoperative complexity in major emergency abdominal surgery: a Danish nationwide survey. Eur Surg 57:11–20. 10.1007/s10353-024-00847-w

[16] Nassar AHM, Hodson J, Ng HJ, Vohra RS, Katbeh T, Zino S et al (2020) Predicting the difficult laparoscopic cholecystectomy: development and validation of a pre-operative risk score using an objective operative difficulty grading system. Surgical endoscopy. Germany: choles study group. West Midlands Res Collaborative 34:4549–4561. 10.1007/s00464-019-07244-5 10.1007/s00464-019-07244-5 31732855

[17] Ramírez-Giraldo C, Alvarado-Valenzuela K, Isaza-Restrepo A, Navarro-Alean J (2022) Predicting the difficult laparoscopic cholecystectomy based on a preoperative scale. Updates Surg Italy 74:969–977. 10.1007/s13304-021-01216-y 10.1007/s13304-021-01216-y PMC9213361 35122205

[18] Kawaguchi Y, Fuks D, Kokudo N, Gayet B (2018) Difficulty of laparoscopic liver resection: proposal for a new classification. Ann Surg 267:13–17. 10.1097/SLA.0000000000002176 28187043 10.1097/SLA.0000000000002176

[19] Muangkaew P, Cho JY, Han H-S, Yoon Y-S, Choi Y, Jang JY et al (2016) Defining surgical difficulty according to the perceived complexity of liver resection: validation of a complexity classification in patients with hepatocellular carcinoma. Ann Surg Oncol 23:2602–2609. 10.1245/s10434-015-5058-2 26727918 10.1245/s10434-015-5058-2

[20] Bourgouin S, Mancini J, Monchal T, Calvary R, Bordes J, Balandraud P (2016) How to predict difficult laparoscopic cholecystectomy? Proposal for a simple preoperative scoring system. Am J Surg United States 212:873–881. 10.1016/j.amjsurg.2016.04.003 10.1016/j.amjsurg.2016.04.003 27329073

[21] Hu ASY, Menon R, Gunnarsson R, de Costa A (2017) Risk factors for conversion of laparoscopic cholecystectomy to open surgery - A systematic literature review of 30 studies. Am J Surg United States 214:920–930. 10.1016/j.amjsurg.2017.07.029 10.1016/j.amjsurg.2017.07.029 28739121

[22] Maehira H, Kawasaki M, Itoh A, Ogawa M, Mizumura N, Toyoda S et al (2017) Prediction of difficult laparoscopic cholecystectomy for acute cholecystitis. J Surg Res United States 216:143–148. 10.1016/j.jss.2017.05.008 10.1016/j.jss.2017.05.008 28807199

[23] Pothet C, Drumez É, Joosten A, Genin M, Hobeika C, Mabrut J-Y et al (2021) Predicting intraoperative difficulty of open liver resections: the DIFF-scOR Study, an analysis of 1393 consecutive hepatectomies from a French multicenter cohort. Ann Surg 274:805–813. 10.1097/SLA.0000000000005133 34353987 10.1097/SLA.0000000000005133

[24] Parker MC, Wilson MS, Menzies D, Sunderland G, Clark DN, Knight AD et al (2005) The SCAR-3 study: 5-year adhesion-related readmission risk following lower abdominal surgical procedures. Colorectal disease: the official journal of the association of coloproctology of great Britain and Ireland. England: surgical and clinical adhesions research (SCAR). Group 7:551–558. 10.1111/j.1463-1318.2005.00857.x 10.1111/j.1463-1318.2005.00857.x 16232234

[25] ten Broek RPG, Strik C, Issa Y, Bleichrodt RP, van Goor H (2013) Adhesiolysis-related morbidity in abdominal surgery. Annals of surgery. United States 258:98–106. 10.1097/SLA.0b013e31826f4969 10.1097/SLA.0b013e31826f4969 23013804

[26] Mavros MN, Bohnen JD, Ramly EP, Velmahos GC, Yeh DD, de Moya M et al (2015) Intraoperative adverse events: risk adjustment for procedure complexity and presence of adhesions is crucial. J Am Coll Surg United States 221:345–353. 10.1016/j.jamcollsurg.2015.03.045 10.1016/j.jamcollsurg.2015.03.045 26141463

[27] von Elm E, Altman DG, Egger M, Pocock SJ, Gøtzsche PC, Vandenbroucke JP (2008) The strengthening the reporting of observational studies in epidemiology (STROBE) statement: guidelines for reporting observational studies. J Clin Epidemiol United States: STROBE Initiative 61:344–349. 10.1016/j.jclinepi.2007.11.008 10.1016/j.jclinepi.2007.11.008 18313558

[28] Peden CJ, Aggarwal G, Aitken RJ, Anderson ID, Bang Foss N, Cooper Z et al (2021) Guidelines for perioperative care for emergency laparotomy enhanced recovery after surgery (ERAS) society recommendations: part 1-Preoperative: Diagnosis, rapid assessment and optimization. World J Surg United States 45:1272–1290. 10.1007/s00268-021-05994-9 10.1007/s00268-021-05994-9 PMC8026421 33677649

[29] Peden CJ, Aggarwal G, Aitken RJ, Anderson ID, Balfour A, Foss NB et al (2023) Enhanced recovery after surgery (ERAS^®^) society consensus guidelines for emergency laparotomy part 3: organizational aspects and general considerations for management of the emergency laparotomy patient. World J Surg United States 47:1881–1898. 10.1007/s00268-023-07039-9 10.1007/s00268-023-07039-9 PMC10241556 37277506

[30] Scott MJ, Aggarwal G, Aitken RJ, Anderson ID, Balfour A, Foss NB et al (2023) Consensus guidelines for perioperative care for emergency laparotomy enhanced recovery after surgery (ERAS(^®^)) society recommendations part 2-Emergency laparotomy: Intra- and postoperative care. World J Surg United States 47:1850–1880. 10.1007/s00268-023-07020-6 10.1007/s00268-023-07020-6 PMC10241558 37277507

[31] Kokotovic D, Burcharth J (2023) Enhanced recovery after emergency laparotomy. The British journal of surgery. England 110:538–540. 10.1093/bjs/znad056 10.1093/bjs/znad056 36896630

[32] Kokotovic D, Jensen TK (2023) Acute abdominal pain and emergency laparotomy: bundles of care to improve patient outcomes. Br J Surg Engl. 10.1093/bjs/znad224 10.1093/bjs/znad224 37449877

[33] Tolstrup M-B, Jensen TK, Gögenur I (2023) Intraoperative surgical strategy in abdominal emergency surgery. World J Surg United States 47:162–170. 10.1007/s00268-022-06782-9 10.1007/s00268-022-06782-9 36221004

[34] Zühlke HV, Lorenz EM, Straub EM, Savvas V (1990) [Pathophysiology and classification of adhesions]. Langenbecks Arch Chir Suppl II Verh Dtsch Ges Chir 1009–1016 https://pubmed.ncbi.nlm.nih.gov/1983476/1983476

[35] Bohnen JD, Mavros MN, Ramly EP, Chang Y, Yeh DD, Lee J et al (2017) Intraoperative adverse events in abdominal surgery: what happens in the operating room does not stay in the operating room. Annals of surgery. United States 265:1119–1125. 10.1097/SLA.0000000000001906 10.1097/SLA.0000000000001906 27805961

[36] Kirchhoff P, Clavien P-A, Hahnloser D (2010) Complications in colorectal surgery: risk factors and preventive strategies. Patient safety in surgery. England 4:5. 10.1186/1754-9493-4-5 10.1186/1754-9493-4-5 PMC2852382 20338045

[37] Daley BJ, Cecil W, Clarke PC, Cofer JB, Guillamondegui OD (2015) How slow is too slow? Correlation of operative time to complications: an analysis from the Tennessee surgical quality collaborative. J Am Coll Surg 220:550–558. 10.1016/j.jamcollsurg.2014.12.040 25728140 10.1016/j.jamcollsurg.2014.12.040

[38] Carter M, Chen AR, Pitt JB, Hua R, Wafford QE, Manworren RC et al (2025) Classification systems of surgical complexity: A scoping review of the literature. J Surg Res 306:570–579. 10.1016/j.jss.2024.12.049 39892301 10.1016/j.jss.2024.12.049

[39] Subcommittee ATLS, American College of Surgeons’ Committee on Trauma, International ATLS working group (2013) Advanced trauma life support (ATLS^®^): the ninth edition. J Trauma Acute Care Surg 74:1363–1366. 10.1097/TA.0b013e31828b82f5 23609291 10.1097/TA.0b013e31828b82f5

[40] Kosaka H, Satoi S, Kono Y, Yamamoto T, Hirooka S, Yamaki S et al (2022) Estimation of the degree of surgical difficulty anticipated for pancreatoduodenectomy: preoperative and intraoperative factors. J Hepatobiliary Pancreat Sci 29:1166–1174. 10.1002/jhbp.1052 34596977 10.1002/jhbp.1052

[41] Slankamenac K, Graf R, Barkun J, Puhan MA, Clavien P-A (2013) The comprehensive complication index: a novel continuous scale to measure surgical morbidity. Annals Surg United States 258:1–7. 10.1097/SLA.0b013e318296c732 10.1097/SLA.0b013e318296c732 23728278

[42] Dindo D, Demartines N, Clavien P-A (2004) Classification of surgical complications: a new proposal with evaluation in a cohort of 6336 patients and results of a survey. Annals Surg United States 240:205–213. 10.1097/01.sla.0000133083.54934.ae 10.1097/01.sla.0000133083.54934.ae PMC1360123 15273542

[43] Schack A, Ekeloef S, Ostrowski SR, Gögenur I, Burcharth J (2022) Blood transfusion in major emergency abdominal surgery. European journal of trauma and emergency surgery: official publication of the European trauma society. Germany 48:121–131. 10.1007/s00068-020-01562-3 10.1007/s00068-020-01562-3 33388785

[44] Halabi WJ, Jafari MD, Nguyen VQ, Carmichael JC, Mills S, Pigazzi A et al (2013) Blood transfusions in colorectal cancer surgery: incidence, outcomes, and predictive factors: an American college of surgeons National surgical quality improvement program analysis. Am J Surg United States 206:1023–1024. 10.1016/j.amjsurg.2013.10.001 10.1016/j.amjsurg.2013.10.001 24296103

[45] Matsuyama T, Iranami H, Fujii K, Inoue M, Nakagawa R, Kawashima K (2013) Risk factors for postoperative mortality and morbidities in emergency surgeries. J Anesth 27:838–843. 10.1007/s00540-013-1639-z 23700220 10.1007/s00540-013-1639-z

[46] Hébert PC, Wells G, Blajchman MA, Marshall J, Martin C, Pagliarello G et al (1999) A multicenter, randomized, controlled clinical trial of transfusion requirements in critical care. Transfusion requirements in critical care Investigators, Canadian critical care trials group. N Engl J Med 340:409–417. 10.1056/NEJM199902113400601 9971864 10.1056/NEJM199902113400601

[47] American Society of Anesthesiologists Task Force on Perioperative Blood Management (2015) Practice guidelines for perioperative blood management: an updated report by the American society of anesthesiologists task force on perioperative blood Management*. Anesthesiology 122:241–275. 10.1097/ALN.0000000000000463 25545654 10.1097/ALN.0000000000000463

[48] Baker L, Park L, Gilbert R, Ahn H, Martel A, Lenet T et al (2021) Intraoperative red blood cell transfusion Decision-making: A systematic review of guidelines. Ann Surg 274:86–96. 10.1097/SLA.0000000000004710 33630462 10.1097/SLA.0000000000004710

[49] Cheng H, Chen BP-H, Soleas IM, Ferko NC, Cameron CG, Hinoul P (2017) Prolonged operative duration increases risk of surgical site infections: A systematic Review. surgical infections. United States 18:722–735. 10.1089/sur.2017.089 10.1089/sur.2017.089 PMC5685201 28832271

[50] Cheng H, Clymer JW, Po-Han Chen B, Sadeghirad B, Ferko NC, Cameron CG et al (2018) Prolonged operative duration is associated with complications: a systematic review and meta-analysis. J Surg Res 229:134–144. 10.1016/j.jss.2018.03.022 29936980 10.1016/j.jss.2018.03.022

[51] Ouaïssi M, Gaujoux S, Veyrie N, Denève E, Brigand C, Castel B et al (2012) Post-operative adhesions after digestive surgery: their incidence and prevention: review of the literature. J Visc Surg France 149:e104–e114. 10.1016/j.jviscsurg.2011.11.006 10.1016/j.jviscsurg.2011.11.006 22261580

