# Supplementary material for: Intraoperative complexity markers are associated with morbidity but not mortality in emergency abdominal surgery: a two-year cohort study
Source: Langenbecks Arch Surg. 2026 Jan 16;411(1):66. doi: 10.1007/s00423-025-03941-z (PMC12847177; doi:10.1007/s00423-025-03941-z)
Supplement: Supplementary file 1 — Supplementary Material 1 [file 423_2025_3941_MOESM1_ESM.docx]

**Supplementary Figures**

**Supplementary Figure 1:** Distribution of intraoperative complexity markers

**Supplementary Figure 2:** Distribution of comprehensive complication Index (CCI) scores by surgical complexity group

**Supplementary Figure 1:** Distribution of intraoperative complexity markers


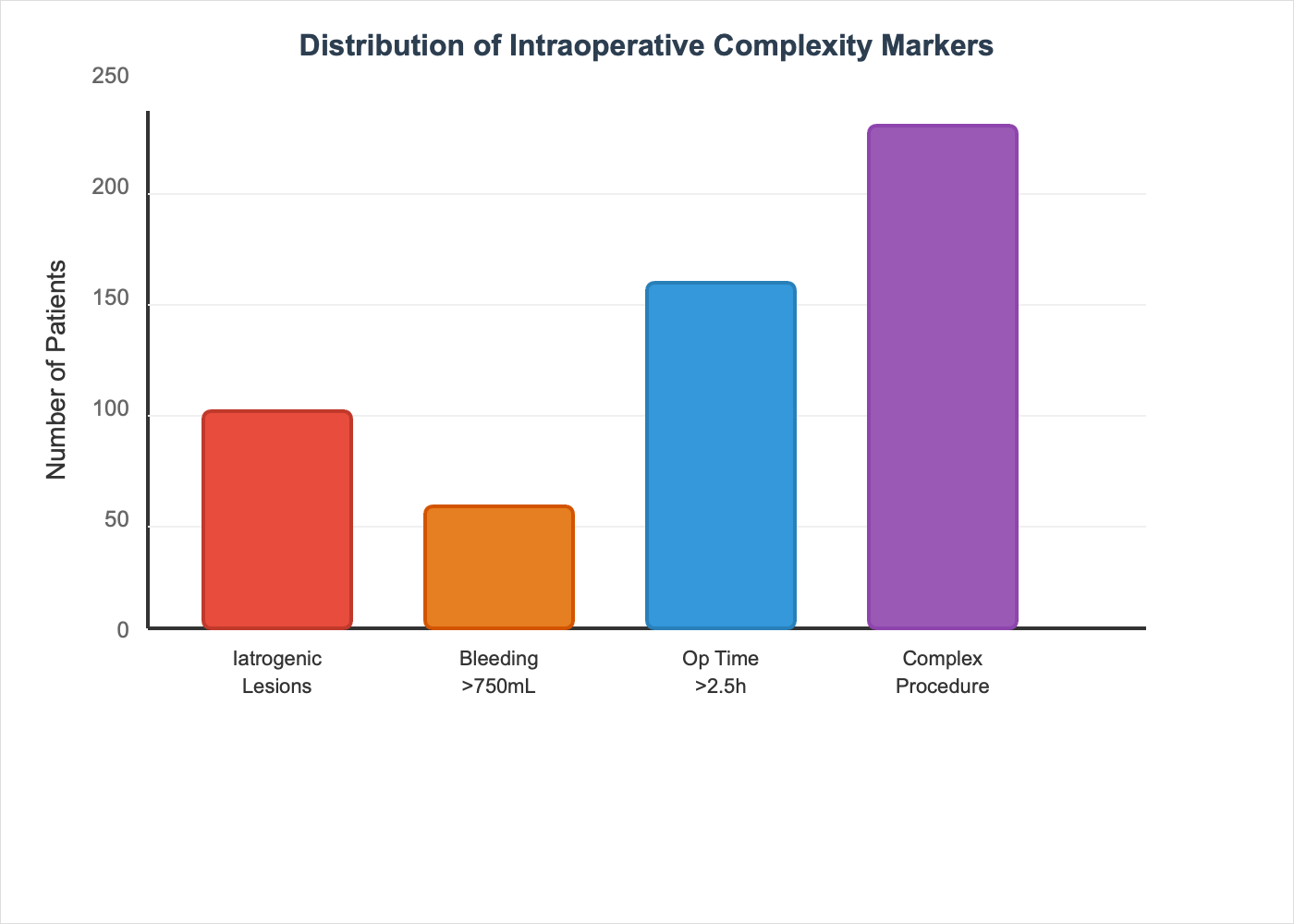


Bar chart showing the frequency of individual complexity markers and composite complexity in 754 consecutive patients undergoing major emergency abdominal surgery. Iatrogenic lesions and operative time >2.5 hours each occurred in 14% and 22% of procedures, respectively, while intraoperative bleeding >750 mL was less common (7%). Overall, 32% of procedures had at least one complexity marker.

**Supplementary Figure 2:** Distribution of comprehensive complication index (CCI) scores by surgical complexity group


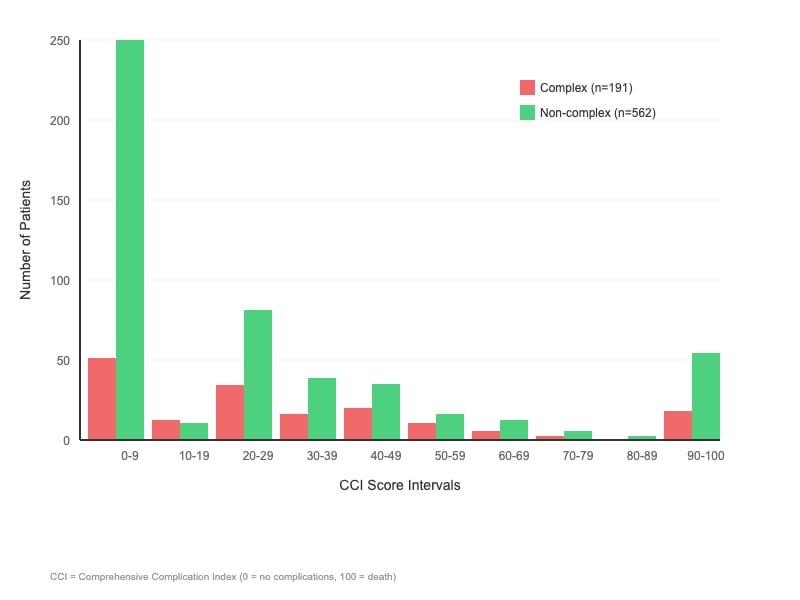


Histogram showing the frequency distribution of CCI scores across 10-point intervals for complex (red, n=191) and non-complex (green, n=562) surgical cases. The CCI ranges from 0 (no complications) to 100 (death), based on the Clavien-Dindo classification system. Complex cases demonstrated a more uniform distribution across all CCI intervals, while non-complex cases showed clustering at lower CCI scores. The median CCI was significantly higher in the complex group (24.2) compared to the non-complex group (12.2), (p<0.001). This distribution pattern explains the linear appearance of the cumulative percentage curves observed in complex cases, reflecting a genuine biological difference in complication severity patterns between surgical complexity groups.
