# Supplementary material for: Intraoperative complexity markers are associated with morbidity but not mortality in emergency abdominal surgery: a two-year cohort study
Source: Langenbecks Arch Surg. 2026 Jan 16;411(1):66. doi: 10.1007/s00423-025-03941-z (PMC12847177; doi:10.1007/s00423-025-03941-z)
Supplement: Supplementary file 2 — Supplementary Material 2 [file 423_2025_3941_MOESM2_ESM.docx]

**Supplementary Material 1**: The AHA protocol at Department of Hepatic and Gastrointestinal Diseases, Copenhagen University Hospital - Herlev and Gentofte, Herlev, Denmark

The following text is a translation of our internal instructions, which are regularly updated to ensure evidence-based solutions and organizational efficiency. The protocol was developed by surgeons, anesthesiologists, and radiologists. The protocol is based on international guidelines and validated bundle-care programs for emergency abdominal surgery [1–5].

**Purpose**

**Target Audience and Scope of Application**

Healthcare personnel at Herlev and Gentofte Hospital.

The guidance applies to all adult emergency gastrointestinal surgical patients who present at the Emergency Department or are admitted to the clinical ward at Herlev and Gentofte Hospital.

**Definitions**

The AHA pathway involves patients suspected of having an acute high-risk gastrointestinal surgical condition that requires urgent surgical intervention, such as exploratory laparotomy or laparoscopy. This includes conditions such as perforated hollow organs, ileus, and ischemic bowel. The AHA pathway does not include appendectomy or laparoscopic cholecystectomy.

**Subacute High-Risk Abdominal Surgery (Subacute-AHA)**: Patients suspected of ileus, where it is deemed that the patient can tolerate and benefit from delaying surgery until the next day. Examples of patients in this group include those who have undergone multiple surgeries with extensive adhesions or cancer patients with disseminated disease, who are not septic or peritoneal. All other optimization measures should be offered – see exceptions below.

**NELA Score**: Used for risk stratification of gastrointestinal surgical patients, where the predicted 30-day mortality is calculated. Use the mobile app (see below). This is entered by the anesthesiologist.

**Procedure**

Upon suspicion of a condition that may require major emergency gastrointestinal surgery, the AHA pathway is initiated by a surgical doctor from the Department of Gastrointestinal and Liver Diseases. The patient should fast and refrain from drinking fluids. It is emphasized that it is not critical if the patient is clinically unwell at triage, but rather if there is suspicion or confirmed abdominal catastrophe that could potentially threaten the patient’s condition. The time factor is crucial, so all steps should be performed as quickly as possible to ensure that early surgical intervention is not delayed.

**Description of the Patient Pathway**

**Emergency Department: Suspicion and Inclusion**

It is aimed that patients suspected of AHA are seen within 30 minutes of arrival by:

• **Acute Department Flowmaster (surgical senior resident)** weekdays 08:00-16:00

• **Surgical on-call** 16:00-08:00 and weekends/holidays

**Arrival at the Emergency Department (FAM)**

Fasting, IV access (at least one green cannula), blood sampling (including type + BAC test and venous blood gas for GFR calculation) should be prioritized as soon as possible before CT scanning.

If the patient is septic (score ≥ 2: temperature >38°C or <36°C, heart rate >90, respiratory rate >20, or leukocytes <4 or >12), or scores on the SOFA score (BP <100, respiratory rate >22, or GCS <14), initiate PIP/Tazo (or Cefuroxime 1.5g if penicillin allergic).

Venous blood gas should be taken for eGFR calculation based on creatinine.

Order **CT-AHA**. CT-AHA is prioritized over other CT scans and takes precedence in the queue. The scan must be completed within 30 minutes.

If necessary:

• Place gastric tube for suction

• Oxygen via nasal cannula

• Fluid treatment

• Pain management

**Time is crucial in this pathway**. At every transition in the patient pathway, healthcare personnel should ensure that care or treatment tasks from the previous location that have not been completed are performed.

**Subacute AHA:**

Subacute high-risk abdominal surgery (subacute-AHA) patients do not require a CT-AHA but can undergo a regular CT scan if it is clinically assessed that early surgery is not immediately beneficial.

**Radiological Diagnostics**

The gastrointestinal surgeon or doctor in the clinical department orders **CT-AHA (abdomen)** in EPIC and contacts the radiologist.

**After CT scan:**

The gastrointestinal surgeon (surgical FAM senior resident or surgical middle shift) will immediately confer with the radiologist about the indication for surgery. Meanwhile, the patient is returned to their bed in FAM. If the patient comes from the clinical department, they will wait for clarification at the CT scanner in FAM.

If the CT scan shows an indication for emergency surgery, the patient is prepared for surgery in FAM. If the patient is from the clinical department, they are transferred to Recovery Room (PACU) for preparation. Surgery must be initiated within 6 hours.

**Preoperative Optimization**

• **TED stockings** applied if indicated.

• **Innohep 3500 IU** ordered.

• **IV antibiotics**: Piperacillin/Tazobactam 4g (if not already administered). For penicillin allergy, give Cefuroxime 1.5g.

• **Metronidazole 1.5g** should be administered if time permits, otherwise it will be administered in Recovery Room (PACU) or in the operating room.

Inform the anesthesiologist and surgical on-call resident about the AHA patient who will be received in Recovery Room (PACU).

The anesthesiologist and gastrointestinal surgeon coordinating the acute surgical bed will prioritize the operation.

**If there is no indication for surgery, the patient will return to the Emergency Department or clinical department.** A decision should be made about discontinuing orders and further planning.

The patient should be prioritized for surgery on the first available operating table and the case should be booked as an AHA procedure.

Conference points:

1. **Treatment limit** (in consultation with anesthesiology on-call)

2. **ASA ≥4** or severe comorbidity/organ dysfunction: Involvement of the anesthesiologist before offering surgery to the patient/family (Final decision on the operation is made by the surgical on-call resident)

3. **Indication for laparoscopic vs laparotomy** and other special considerations

4. **AHA pre-assessment form** should be filled out

5. **Preoperative identification and consent** should be completed

**Subacute AHA:**

For a patient who requires subacute high-risk abdominal surgery, book the patient via the regular AHA pathway, but change the case booking to “AHA subacute.” The patient remains in the ward until surgery is performed.

**Postoperative Course**

The surgeon writes the operative report, including decisions regarding continued use of tubes (for suction or overflow), nutrition plan, pain management, etc. The completion of the peroperative AHA form should be done with planning.

**Recovery: Postoperative Care Unit (PACU)**

Immediately postoperatively, the patient should be NELA scored by the anesthesiologist, and based on the 30-day mortality, the patient should be assigned to a differentiated postoperative pathway via the Perioperative Risk calculator app.

30-day mortality estimated with NELA score and postoperative recovery status:

• **0-4.9%**: Standard recovery

• **5-9.9%**: 12-hour recovery

• **>10%**: 24-hour recovery

Postoperative rounds should be done by anesthesiology and surgical on-call (or their delegated personnel), focusing on:

• Pain management

• Consciousness – including delirium

• Respiratory function (PEEP/cpap)

• Fluid status and fluid management plan

• Antibiotic plan (as indicated in operative report)

• Thrombosis prophylaxis (initiated postoperatively if not started preoperatively)

• For NELA ≥5%: Mobilization to a chair (if possible)

• Nutrition (protein drink if possible)

Upon discharge from Recovery Room (PACU), the anesthesiologist provides status and postoperative plan.

**Subacute AHA:**

Subacute high-risk abdominal surgery patients follow the same postoperative regimen as other AHA patients.

**Inpatient Care**

Referrals should be made according to the diagnosis-specific guidelines and instructions in the Department of Gastrointestinal and Liver Diseases, including mechanical ileus.

The postoperative course involves standardized high-dependency wards, intermediate or intensive care as needed, with daily rounds conducted by dedicated consultant emergency surgeons. A standardized nursing care plan is followed, ensuring optimal pain management, dietician-regulated early nutrition, and physiotherapist-guided mobilization. Regular data audits and quality assessments are performed to ensure the highest standards of care.

**References**

1. Tengberg LT, Bay-Nielsen M, Bisgaard T, Cihoric M, Lauritsen ML, Foss NB. Multidisciplinary perioperative protocol in patients undergoing acute high-risk abdominal surgery. The British journal of surgery. 2017;104:463–71.

2. Huddart S, Peden CJ, Swart M, McCormick B, Dickinson M, Mohammed MA, et al. Use of a pathway quality improvement care bundle to reduce mortality after emergency laparotomy. The British journal of surgery. 2015;102:57–66.

3. Peden CJ, Aggarwal G, Aitken RJ, Anderson ID, Bang Foss N, Cooper Z, et al. Guidelines for Perioperative Care for Emergency Laparotomy Enhanced Recovery After Surgery (ERAS) Society Recommendations: Part 1-Preoperative: Diagnosis, Rapid Assessment and Optimization. World journal of surgery. 2021;45:1272–90.

4. Scott MJ, Aggarwal G, Aitken RJ, Anderson ID, Balfour A, Foss NB, et al. Consensus Guidelines for Perioperative Care for Emergency Laparotomy Enhanced Recovery After Surgery (ERAS(®)) Society Recommendations Part 2-Emergency Laparotomy: Intra- and Postoperative Care. World journal of surgery. 2023;47:1850–80.

5. Peden CJ, Aggarwal G, Aitken RJ, Anderson ID, Balfour A, Foss NB, et al. Enhanced Recovery After Surgery (ERAS®) Society Consensus Guidelines for Emergency Laparotomy Part 3: Organizational Aspects and General Considerations for Management of the Emergency Laparotomy Patient. World journal of surgery. 2023;47:1881–98.
