# Supplementary material for: Intraoperative complexity markers are associated with morbidity but not mortality in emergency abdominal surgery: a two-year cohort study
Source: Langenbecks Arch Surg. 2026 Jan 16;411(1):66. doi: 10.1007/s00423-025-03941-z (PMC12847177; doi:10.1007/s00423-025-03941-z)
Supplement: Supplementary file 3 — Supplementary Material 3 [file 423_2025_3941_MOESM3_ESM.docx]

**Supplementary Tables**

**Supplementary Table 1**: Baseline patient characteristics and preoperative variables.

**Supplementary Table 2**: Intraoperative variables.

**Supplementary Table 3**: Average marginal effects of intraoperative complexity markers on binary postoperative outcomes.

**Supplementary Table 4**: Average marginal effects of intraoperative complexity markers on continuous postoperative outcomes.

**Supplementary Table 5**: ROC analysis of intraoperative bleeding volume as a predictor of postoperative outcomes.

**Supplementary Table 6**: ROC analysis of operative time (minutes) as a predictor of postoperative outcomes.

**Supplementary Table 1**: Baseline patient characteristics and preoperative variables

|  | **Patients,** *N* = 754 |
| --- | --- |
| **Age** in years, median (IQR) | 71 (58 – 79) |
| **Sex**, female (%) | 383 (51) |
| **BMI**^a^, mean (SD) | 25 (± 5) |
| **ASA**^b^ >2 (%) | 303 (40) |
| **PS**^c^ >1 (%) | 194 (26) |
| **Alcohol** (%)  No use  1 – 7  8 – 14  >14  N/A | 161 (21)  302 (40)  174 (23)  77 (10)  40 (5) |
| **Smoking** (%)  Never  Previously or active  N/A | 297 (39)  436 (57)  21 (3) |
| **Comorbidity** (%)  Previous MI^d^ and/or IHD^e^  Cardiac insufficiency  Atrial fibrillation | 66 (9)  20 (3)  91 (12) |
| COPD^f^ | 62 (8) |
| Diabetes Type 1 or 2 | 81 (11) |
| Liver cirrhosis | 25 (3) |
| Chronic kidney disease | 25 (3) |
| Previously stroke  Dementia  **Preoperative medical treatment** (%)  Anticoagulants^g^  Antidiabetics^h^  Chemo-, immunotherapy or steroids | 53 (7)  13 (2)  164 (22)  63 (8)  127 (17) |
| **Cancer disease** (%)  Never  Active cancer  Previously cancer  Active chemotherapy | 476 (63)  117 (16)  116 (15)  39 (5) |
| **Previously abdominal and/or pelvic surgery** (%)  Laparoscopic  Laparoscopic and open  Open | 460 (61)  108 (14)  249 (33)  97 (13) |

Values are number of patients (%) unless stated otherwise. ^a^BMI; Body Mass Index, ^b^ASA; American Society of Anesthesiologists, ^c^PS; Performance score, ^d^MI; Myocardial infarction, ^e^IHD; Ischemic heart disease, ^f^COPD; Chronic obstructive pulmonary disease, ^g^Anticoagulants: vitamin K-antagonists, factor Xa-antagonists, factor IIa-antagonists or platelet inhibitors. ^h^Antidiabetics: Insulin or metformin

**Supplementary Table 2**: Intraoperative variables

|  | **Patients,** *N* = 754 |
| --- | --- |
| **Method of operation** (%)  Laparoscopy  Laparotomy  Laparotomy (converted from laparoscopy) | 160 (21)  386 (51)  208 (28) |
| **Intraoperative pathology** (%)  Bowel obstruction  Perforated viscus  Bowel ischemia  Fascial dehiscence  Anastomotic leakage  Incarcerated hernia  Other* | 445 (59)  205 (27)  47 (6)  5 (1)  9 (1)  18 (2)  124 (16) |
| **Surgical strategy** (%)  Definitive  Palliative  Damage control | 666 (88)  41 (5)  44 (6) |
| **Intraoperative findings** (%)  Intraabdominal contamination, grade 3 – 4  Carcinosis  Adhesions grade 0 – 2  Adhesions, grade 3: Severe adhesions  Adhesions, grade 4: Frozen abdomen  Adhesions, grade 3 – 4 | 201 (27)  42 (6)  624 (83)  92 (12)  21 (3)  113 (15) |

*Bleeding, non-traumatic spleen rupture, intraabdominal abscess, or laparotomy with no pathology found.

**Supplementary Table 3**: Average marginal effects of intraoperative complexity markers on binary postoperative outcomes

| **Complexity Marker** | **n (exposed/total)** | **Clavien-Dindo >3b** | | **Reoperation** | | **LOS >7 days** | | **30-day Mortality** | |
| --- | --- | --- | --- | --- | --- | --- | --- | --- | --- |
|  |  | **AME% (95% CI)** | **p** | **AME% (95% CI)** | **p** | **AME% (95% CI)** | **p** | **AME% (95% CI)** | **p** |
| **Iatrogenic Lesions** | 104/754 | 8.9 (-1.8, 19.6) | 0.10 | **8.8 (0.1, 17.5)** | **0.05** | **12.6 (2.9, 22.3)** | **0.01** | -6.8 (-6.8, 6.4) | 0.95 |
| **Bleeding >750mL** | 56/754 | 7.3 (-7.1, 21.7) | 0.32 | **16.0 (2.1, 29.9)** | **0.02** | **23.0 (8.5, 37.5)** | **0.002** | 7.1 (-2.8, 17.0) | 0.16 |
| **Op Time >2.5h** | 168/754 | 5.8 (-3.1, 14.7) | 0.20 | 7.5 (-0.8, 15.8) | 0.08 | **22.1 (12.8, 31.4)** | **<0.001** | 0.1 (-5.7, 5.9) | 0.98 |
| **Complex Procedure (Any)** | 243/754 | **7.4 (0.2, 14.6)** | **0.04** | **8.3 (1.7, 14.9)** | **0.01** | **17.4 (9.5, 25.3)** | **<0.001** | -1.8 (-6.5, 2.9) | 0.45 |

AME = Average Marginal Effect (percentage points). CI = Confidence Interval. Adjusted for age, sex, ASA>2, COPD, cirrhosis, diabetes, CKD, and previous MI/IHD.

This table presents the adjusted average marginal effects (AMEs) representing percentage point changes in outcome probability associated with each complexity marker after controlling for age, sex, ASA>2, COPD, cirrhosis, diabetes, CKD, and previous MI/IHD. Operating time >2.5 hours strongly associated with prolonged hospitalization, increasing the probability by 22.1 percentage points (95% CI: 12.8-31.4, p<0.001). Notably, no complexity marker significantly increased 30-day mortality after adjustment, with all showing negative point estimates.

**Supplementary Table 4**: Average marginal effects of intraoperative complexity markers on continuous postoperative outcomes

| **Complexity Marker** | **n (exposed/total)** | **CCI Score Mean Difference (95% CI)** | **p** | **Length of Stay Days Difference (95% CI)** | **p** |
| --- | --- | --- | --- | --- | --- |
| **Iatrogenic Lesions** | 104/754 | 5.7 (-0.5, 11.9) | 0.07 | **7.2 (3.7, 10.7)** | **<0.001** |
| **Bleeding >750mL** | 56/754 | 6.4 (-2.8, 15.6) | 0.17 | **9.7 (4.4, 15.0)** | **<0.001** |
| **Op Time >2.5h** | 168/754 | **6.5 (0.6, 12.4)** | **0.03** | **8.9 (5.5, 12.3)** | **<0.001** |
| **Complex Procedure (Any)** | 243/754 | **6.4 (1.6, 11.2)** | **0.009** | **5.7 (3.1, 8.3)** | **<0.001** |

Adjusted mean differences in Comprehensive Complication Index (CCI) scores and length of stay (LOS) associated with complexity markers. All markers significantly prolonged hospitalization after controlling for patient demographics and comorbidities, with bleeding >750 mL showing the largest effect (+9.7 days, 95% CI: 4.4-15.0, p<0.001). Operating time >2.5 hours, and the composite complexity measure increased CCI scores by approximately 6.5 points, demonstrating the substantial impact of intraoperative complexity on postoperative recovery independent of patient factors.

**Supplementary Table 5**: ROC analysis of intraoperative bleeding volume as a predictor of postoperative outcomes.

| Outcome | Optimal cut-off (mL) | Sensitivity | Specificity | AUC |
| --- | --- | --- | --- | --- |
| Length of stay (continuous) | 5.0 | 0.17 | 1.00 | 0.58 |
| Length of stay > 7 days | 22.5 | 0.51 | 0.71 | 0.61 |
| Comprehensive Complication Index | 15.5 | 0.41 | 0.68 | 0.53 |
| Clavien–Dindo > 3a | 27.5 | 0.49 | 0.66 | 0.57 |
| Reoperation | 22.5 | 0.52 | 0.64 | 0.59 |
| 30-day mortality | 1900.0 | 0.04 | 0.98 | 0.48 |

Receiver operating characteristic (ROC) analyses were performed using intraoperative bleeding volume (mL) as the test variable for selected postoperative outcomes. The area under the curve (AUC) values ranged from 0.53 to 0.61, indicating modest but consistent discrimination. The optimal cut-offs identified by Youden’s index were numerically low (20–30 mL) due to the right-skewed distribution, where a large proportion of patients experienced minimal or no measurable blood loss. Such thresholds are not clinically meaningful, as they fall within the range of routine intraoperative oozing rather than true hemorrhage. The predefined threshold of 750 mL was therefore retained, as it reflects the transition to hemodynamically relevant bleeding corresponding to ATLS class II hemorrhage (750–1500 mL), consistent with established surgical and trauma literature.

**Supplementary Table 6**: ROC analysis of operative time (minutes) as a predictor of postoperative outcomes.

| Outcome | Optimal cut-off (min) | Sensitivity | Specificity | AUC |
| --- | --- | --- | --- | --- |
| Length of stay | 91.5 | 0.66 | 0.58 | 0.67 |
| Length of stay > 7 days | 91.5 | 0.66 | 0.58 | 0.67 |
| Comprehensive Complication Index | 90.5 | 0.63 | 0.56 | 0.62 |
| Clavien–Dindo > 3a | 83.5 | 0.64 | 0.44 | 0.55 |
| Reoperation | 156.5 | 0.28 | 0.81 | 0.54 |
| 30-day mortality | 111.5 | 0.74 | 0.39 | 0.54 |
| Mortality during follow-up | 95–100 | 0.60 | 0.51–0.59 | 0.56 |

Receiver operating characteristic (ROC) analyses were performed using operative time (minutes) as the test variable for selected postoperative outcomes. The AUC values ranged from 0.55 to 0.67, demonstrating moderate discriminatory ability across outcomes. Optimal cut-offs identified by Youden’s index clustered between 90 and 160 minutes, corresponding closely to the predefined threshold of 150 minutes (2.5 hours) used in the main analyses. This value represents a clinically meaningful inflection point distinguishing standard from prolonged emergency procedures and is consistent with prior literature linking operative duration beyond 2–3 hours with elevated postoperative morbidity.
